# Thyroid function tests, incongruent internally and with thyroid status, both in a pregnant woman and in her newborn daughter

**DOI:** 10.1530/ETJ-21-0088

**Published:** 2022-04-22

**Authors:** Federica D’Aurizio, Alessia Biasotto, Claudia Cipri, Franco Grimaldi, Jessica Zucco, Stefania Marzinotto, Francesco Curcio, Salvatore Benvenga

**Affiliations:** 1 Department of Laboratory Medicine, Institute of Clinical Pathology, University Hospital of Udine, Udine, Italy; 2 Department of Medicine, University of Udine, Udine, Italy; 3 Endocrinology, Metabolism and Clinical Nutrition Unit, University Hospital of Udine, Udine, Italy; 4 Department of Clinical and Experimental Medicine, Endocrinology Section, University of Messina, Messina, Italy

**Keywords:** thyroid function tests, immunoassay, thyroid disease, pregnancy

## Abstract

**Introduction:**

Thyroid function tests (TFT) are extensively used in daily clinical practice. Here, we described a case of incongruent TFT both in a pregnant woman and in her newborn.

**Case presentation:**

A 32-year-old woman, diagnosed with autoimmune thyroiditis during her first pregnancy, was monitored during her second gestation. At week 5 + 2 days, thyroid-stimulating hormone (TSH) and free thyroxine (FT4) values (Dimension VISTA 1500, Siemens Healthineers) were within normal limits. At week 19 + 5 days, TSH remained normal while FT4 increased approximately by three-fold. FT4 inconsistency was with both TSH and the clinical status since she continued to be clinically euthyroid. On the same serum sample, thyroid autoantibodies were negative. At week 25 + 4 days, the patient complained of palpitations and dyspnea, with tachycardia. Even though TSH was normal, high levels of both FT4 and free triiodothyronine (FT3) were interpreted as evidence of thyroid overactivity and methimazole was started. TFT of the pregnant woman continued to be monitored throughout gestation. Postpartum FT4 and FT3 gradually returned to normal. TFT, performed on the daughter’s serum, 3 days after birth, showed the same inconsistency as her mother but without clinical signs of congenital hyperthyroidism. Based on the clinical and laboratory setting, the presence of circulating autoantibodies against T3 and T4 (THAb) was suspected and demonstrated by radioimmunoprecipitation.

**Conclusion:**

Analytical interferences should be supposed when TFT do not fit with the clinical picture and despite their infrequency, THAb must also be considered. To our knowledge, this is the first case describing the passage of THAb to the newborn.

## Established facts

- Thyroid function tests (TFT) are the most commonly requested endocrine tests in both inpatient and outpatient settings.
- Constant interaction among the clinician, the laboratory and the manufacturer is of paramount importance in order to recognize and avoid pitfalls and caveats in the use of these tests, so that they can be correctly interpreted.

## Novel insights

- The case reported here is, to our knowledge, the first to describe the transplacental passage of circulating autoantibodies against T3 and T4 (THAb) (IgG-T3 and IgG-T4), causing, in the newborn, the same inconsistent TFT pattern of the mother.

## Introduction

Thyroid function tests (TFT) represent a significant portion of the laboratory activity since thyroid diseases are very common worldwide (1, 2).

In most patients, TFT results correctly confirm or exclude classical thyroid function states. However, there are cases of discordant TFT, tracing back to a particular clinical picture or to the use of medications that perturb the hypothalamus–pituitary–thyroid axis (HPT) (3). Sometimes, TFT interpretation is challenging because of discordancy with the patient’s clinical presentation or because one hormone level does not fit with the others (4). In such cases, analytical interferences should be investigated in order to avoid unnecessary further diagnostic procedures and/or treatments (5). In fact, despite numerous advances in immunoassay technology, thyroid hormone determination is still prone to at least six main types of analytical interference: macro thyroid-stimulating hormone (TSH), heterophilic antibodies, anti-streptavidin antibodies, anti-ruthenium antibodies, biotin and antibodies against triiodothyronine (T3) and thyroxine (T4) (THAb) (5, 6).

## Case presentation

A case of incongruent TFT due to THAb both in a pregnant woman and in her newborn is described.

In her previous pregnancy, a 32-year-old woman had been diagnosed with Hashimoto’s thyroiditis, associated with subclinical hypothyroidism (thyroperoxidase autoantibodies, TPOAb = 74 U/mL, cut-off = 60 U/mL, Advia Centaur XP, Siemens Healthineer). Accordingly, she had been treated with levothyroxine and stopped 6 months after the delivery of a healthy baby, coinciding with normalization of TPOAb concentrations.

Because of her history, TFT were monitored during her second gestation. At week 5 + 2 days, TSH and FT4 values were normal and congruent (1.69 mIU/L, reference interval: 0.36–3.74 mIU/L; 15.3 pmol/L, reference interval: 9.8–18.8 pmol/L, respectively; Dimension VISTA 1500, Siemens Healthineers) (Table 1).

**Table 1:** Serum TSH, FT4 and FT3 values of the mother (during pregnancy and postpartum) and the daughter with different immunoassays.

Sample Timing	TSH (mUI/L)	FT4 (pmol/L)	FT3 (pmol/L)	Analytical platform	FT4/FT3 Assay	Therapy
5 weeks + 2 days	1.69 (0.36–3.74)	15.3 (9.8–18.8)		Dimension Vista 1500	LOCI, homogeneous	-
19 weeks + 5 days	1.50 (0.36–3.74)	**41.6** (9.8–18.8)		Dimension Vista 1500	LOCI, homogeneous	-
25 weeks + 4 days	1.48 (0.36–3.74)	**39.7** (9.8–18.8)	** 15.2** (3.3–6.1)	Dimension Vista 1500	LOCI, homogeneous	MMI 10 mg/day
28 weeks + 1 day	1.10 (0.36–3.74)	**35.9** (9.8–18.8)	**15.5** (3.3–6.1)	Dimension Vista 1500	LOCI, homogeneous	MMI 10 mg/day
3 days Pp	**4.70** (0.36–3.74)	**24.97** (9.8–18.8)	**11.83** (3.3–6.1)	Dimension Vista 1500	LOCI, homogeneous	Stop MMI
*3 days (daughter)*	*4.68* (*0.82*–*5.91)*^a^	*58.7* (*11.3*–*19.1*)^a^	*15.7* (*5.1*–*8.1*)^a^	*Dimension Vista 1500*	LOCI, homogeneous	
41 days Pp	1.30 (0.36–3.74)	**25.6** (9.8–18.8)	**12.8** (3.3–6.1)	Dimension Vista 1500	LOCI, homogeneous	-
	2.06 (0.27–4.20)	15.9 (12.0–22.0)	4.6 (3.1–6.8)	Cobas e601	ECLIA, one step with two sequential incubations	
	1.57 (0.30–4.00)	12.1 (7.7–15.4)	5.3 (3.8–6.0)	UniCel DxI 800	CLIA, two-step	
*41 days (daughter)*	*1.10 (0.82–5.91)*	*24.7 (11.3–19.1)*	*12.4 (5.1–8.1)*	*Dimension Vista 1500*	*LOCI, homogeneous*	
	*1.64* (*0.72*–*11.00*)^b^	*18.7* (*11.5*–*28.3*)^b^	*7.2* (*3.0*–*9.3*)^b^	Cobas e601	ECLIA, one-step with two sequential incubations	
	*1.12 (0.79–5.85)* (12)	*14.3* (*9.5*–*17.8*) (12)	*6.0 (4.3–6.8)* (12)	UniCel DxI 800	CLIA, two-step	
7 months Pp	1.69 (0.36–3.74)	16.4 (9.8–18.8)	**8.6** (3.3–6.1)	Dimension Vista 1500	LOCI, homogeneous	-
18 months Pp	1.40 (0.36–3.74)	14.7 (9.8–18.8)	4.6 (3.3–6.1)	Dimension Vista 1500	LOCI, homogeneous	

Data of the daughter are given in italics. Hormone levels outside the reference intervals (in brackets) are given in bold.

^a^Reference intervals for infants (≤23 months) as declared by manufacturer’s insert. ^b^Reference intervals for infants (>6 days or ≤3 months) as declared by manufacturer’s insert.

CLIA, chemiluminescence immunoassay; ECLIA, electrochemiluminescence immunoassay; LOCI, luminescent oxygen channeling immunoassay; MMI, methimazole; Pp, postpartum.

At week 19 + 5 days, TFT were repeated but results were inconsistent. While TSH remained normal (1.50 mIU/L), FT4 increased approximately by three-fold (41.6 pmol/L) (Table 1). FT4 was inconsistent with both TSH and the clinical status since she continued to be clinically euthyroid. On the same serum sample, thyroid autoantibodies were negative (TPOAb = 52 U/mL; thyroglobulin antibodies, TgAb = 17 U/mL, cut-off = 60 U/mL, Advia Centaur XP, Siemens Healthineer; anti-TSH receptor antibodies, TRAb = 0.08 IU/L, cut-off = 0.4 IU/L, ElisaRSRTM TRAb third generation). No therapy was started.

At week 25 + 4 days, the patient complained of palpitations and dyspnea, with tachycardia noticed at physical examination. Such symptoms, suggestive of hyperthyroidism/thyrotoxicosis, prompted a further evaluation of TFT. Although TSH continued to be normal, high levels of free thyroid hormones (FT4 = 39.7 pmol/L; FT3 = 15.2 pmol/L) were interpreted as evidence of thyroid overactivity (Table 1). Thus, methimazole therapy (10 mg/day) was initiated. The woman was followed by monitoring TFT for the remainder of the pregnancy (Table 1), which continued without further clinical problems until the full-term birth of a healthy girl.

At 3 postpartum days, the daughter's TFT showed the same inconsistent pattern of the mother with normal TSH and elevated free thyroid hormones (TSH = 4.68 mIU/L; FT4 = 58.7 pmol/L; FT3 = 15.7 pmol/L) (Table 1); however, the baby presented no clinical signs of congenital hyperthyroidism.

On the advice of the endocrinologist who dealt with the clinical case after delivery, laboratory investigations were performed on serum samples collected from both mother and daughter 41 days after childbirth. First, in order to exclude the autosomal dominant familial dysalbuminemic hyperthyroxinemia (FDH), characterized by normal TSH with often falsely elevated serum thyroid hormone levels determined by different automated immunoassays (7), albumin sequencing was performed. Genomic DNA of the adult patient was extracted from whole blood using the QIAsymphony DSP DNA mini kit (Qiagen) and the whole of exon 7 was subsequently amplified using the FW 5’-TCTACCTACCACACACACTCT-3’ and RW 5’-TCTACCAACTTGAGCATGCA-3’ primers; cycling conditions were as follows: 95°C for 5 min, 35 cycles of 95°C for 30 s, 60°C for 30 s, 72°C for 30 s and a final extension at 72°C for 5 min. The PCR product of 387 bp was confirmed by gel electrophoresis, purified by Illustra ExoProStar 1-step digestion and subsequently subjected to bidirectional sequencing (8, 9).

**Figure 1:**
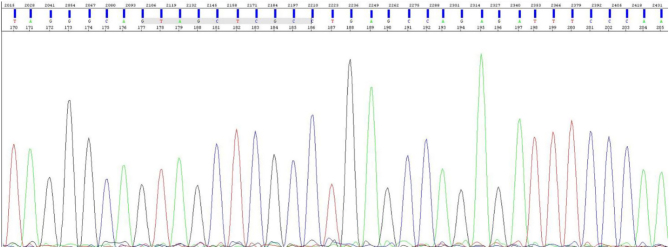
Sanger sequencing chromatogram of the whole exon 7 in the mother’s albumin gene. The analyzed region was found to be wild-type.

As shown in Fig. 1, the mother did not display any genetic variant in the analyzed region.

To better evaluate the TFT pattern, serum total triiodothyronine (TT3) and total thyroxine (TT4) of both the mother and the daughter were measured by Liaison XL (DiaSorin S.p.A., Saluggia, Italy) obtaining normal values (TT3 = 1.42 and 2.50 nmol/L for the mother and the daughter, respectively; RI: 1.18–3.40; TT4 = 78.33 and 90.30 nmol/L for the mother and the daughter, respectively; RI: 57.6–161.3 nmol/L). These results helped to rule out the FDH hypothesis as this autosomal dominant disease is characterized by high serum levels of TT4 as well as FT4 (9).

Based on these findings, the presence of analytical interference was highly suspected; thus, first, serum rheumatoid factor, total immunoglobulin (Ig)G, total IgA, total IgM and antinuclear antibodies (ANA) were measured with all values within the corresponding normal range (data not shown). Rheumatoid factor, total IgM, total IgA and total IgG were determined with immunonephelometric method on Dimension Vista 1500 (Siemens Healthineers) (cut-off = 10 IU/mL; reference intervals: 40–230, 70–400 and 700–1600 mg/dL, respectively); ANA were measured by indirect immunofluorescence (Inova Diagnostics, Werfen Company, San Diego, CA, USA) (cut-off = 1:80).

The presence of heterophilic antibodies was considered unlikely because most frequently described in the literature for TSH measurement (10). Instead, the most likely explanation was an analytical interference on FT3 and FT4 measurements, caused by circulating THAb. Because such interference is exerted only in some immunoassay kits (11), to demonstrate this hypothesis, TFT of both the mother and the daughter were performed on two other analytical systems (Cobas e601, Roche Diagnostics and UniCel DxI 800, Beckman Coulter) (12). The laboratory results were within the normal range, consistent with the normality of serum TSH and clinical status, in sharp contrast with the very high values measured by Dimension Vista 1500 LOCI assay (Table 1).

THAb were searched in the serum from both the mother and her daughter using a radioimmunoprecipitation technique, described in detail in previously published studies (10, 13, 14). In brief, the tested serum is left to equilibrate with tracer amounts of either ^125^I-T3 or ^125^I-T4. Serum preloaded with radioactive T3 or T4 is then left to equilibrate with anti-human IgM or anti-human IgG and then precipitated with polyethylene glycol followed by centrifugation to separate specifically IgM-bound and/or IgG-bound T3 or T4 from free (unbound) T3 and T4. Results are expressed as percent radioactivity precipitated over total radioactivity added.

Circulating THAb were detected in both the mother with the triple pattern IgM-T3 (9%, cut-off = 3.9%), IgG-T3 (5%, cut-off = 3.6%) and IgG-T4 (6%, cut-off = 3.4%) and the daughter with the double pattern IgG-T3 (4.2%, cut-off = 3.6%) and IgG-T4 (5%, cut-off = 3.4%). Because IgG, but not IgM, crosses the placenta and because IgG-T3 and IgG-T4 are shared by the mother and her daughter, we concluded that the presence of IgG-T3 and IgG-T4 in the newborn daughter reflected transplacental passage of maternal IgG-T3 and IgG-T4.

A reevaluation of TFT was performed for the mother at 7 months postpartum with FT4 returning to normal limits (16.4 pmol/L) and a mild persistence of FT3 elevation (8.6 pmol/L). Furthermore, as was to be expected, FT3 also returned perfectly within the normal reference intervals (4.6 pmol/L) at 18 months after the birth of the daughter (Table 1).

## Discussion

TSH is recommended as the first-line test in population screening since it is a reliable hormone in describing thyroid status due to the inverse logarithmic relationship with FT4 if the HPT axis is preserved (1, 2). In most of the cases, where the use of FT4 and rarely FT3 are necessary to complement the clinical workup, TFT patterns are consistent or otherwise easily explained by the patient's clinical condition and/or the drug assumption. Only a minority of TFT inconsistencies can be attributable to immunoassay analytical interference (3, 4, 5).

We described the case of a pregnant woman with normal TSH value and, conversely, elevated FT3 and FT4 concentrations. The discordant TFT pattern persisted throughout the pregnancy and for a few months after delivery. The same pattern was present in the daughter at birth and up to 41 days after delivery.

The differential diagnosis for elevated serum levels of free thyroid hormones coexisting with normal serum levels of TSH comprises drug assumption (e.g. thyroxine and amiodarone), pathological conditions (e.g. TSH-secreting pituitary adenoma, resistance to thyroid hormone, disorders of thyroid hormone transport or metabolism and non-thyroidal illness) and drug interference (e.g. biotin consumption) (3, 4, 5, 6).

Another possible cause of normal TSH levels accompanied by increased thyroid hormone concentrations (T3 and T4) is FDH, which was eventually ruled out in this case, based on three main observations: the normal values of total thyroid hormones, the sudden increase in serum concentrations of thyroid hormones with returning to normal after childbirth and the absence of two of the possible mutations in albumin gene (residue R242 and R222I in exon 7), described in FDH (7, 8, 9).

Thus, based on the medical history and the clinical picture of the mother and daughter, analytical interference was suspected and then supported by various analytical investigations including the absence of interference with two other methods and, in the end, the demonstration of the presence of anti-T3 and anti-T4 antibodies.

THAb are autoantibodies directed against iodinated epitopes of thyroglobulin (15). In particular, thyroglobulin epitopes for THAb can be identified at positions 20–190, 1277–1314 and 2403–2768 as recently described (15). Although the prevalence of THAb in the general population is low (from less than 1.0 to 1.8%) (16, 17, 18), they are more frequent in patients with autoimmune diseases, in particular with autoimmune thyroid disease (18, 19). However, this frequency may be underestimated because THAb are not routinely evaluated and they are often transient in serum. Beyond the extent of their prevalence, THAb interference, in some routine thyroid hormone measurements, is less than 2% of the cases testing positive, mainly because of their different specificity and affinity to the assay antibodies (10, 11). Theoretically, THAb affect only one-step assays, characterized by physical contact between the tracer analog and the patient’s hormone: anti-T3 and anti-T4 antibodies may bind to the labeled hormone analog, reducing the final signal and so producing a falsely elevated hormone value (10, 20). However, in practice, some one-step immunoassays are unresponsive to THAb, and, on the other hand, few two-step immunoassays could be affected by THAb. Probably, the different elements, making up the design of the assay, may influence the sensitivity to the interfering antibodies (20, 21). In the case described here, the discordant pattern obtained with Siemens Dimension Vista 1500 (a homogeneous, chemiluminescent immunoassay based on LOCI® technology) could be due to the greater sensitivity of one-step methods to THAb interference; conversely, TFT were consistent when measured by a two-step immunoassay on Beckman Coulter UniCel DxI 800.

However, the third method on Cobas e601 platform (Roche Diagnostics) confirms the role of the different elements making up the assay architecture in determining its sensitivity to THAb. In fact, it gives no interference, despite being a one-step method, probably because it involves two sequential incubation steps: first, the serum and a ruthenium-labeled capture antibody are mixed, then the biotin-labeled T4 analog that binds the remaining free sites on the capture antibody is added. Undoubtedly, equilibrium dialysis, not routinely available in laboratories, should be used as a reference method (5).

The presence of serum THAb in a pregnant woman, both IgG and IgM, is considered a rare situation. In a recent study on 412 pregnant women tested at 7–11 weeks of gestation, the prevalence of THAb was 5.1%, almost four-fold less than the prevalence of rates of TPOAb and/or TgAb positivity (18.6%) (22). The pathogenesis of THAb in pregnancy is not clear. Undoubtedly, pregnancy is a particular condition triggering autoimmune manifestations (23). In our case, the woman had developed a presumed autoimmune subclinical hypothyroidism in the first gestation. During the second pregnancy and the postpartum period, the assessment of the THAb trend supports the pregnancy-induced autoimmune hypothesis; in fact, after delivery, even with the analytical platform previously sensitive to THAb interference, the values of thyroid hormones decreased until they were almost within normal limits; the persistence of a slight increase in FT3 could be explained by the fact that FT3 immunoassays are known to be more sensitive to analytical interference than FT4 (3, 4, 5).

To our knowledge, there is only another similar experience in literature (10). In that previous case report, THAb started to be detected at week 26 of pregnancy. Based on the lack of false overestimation of both FT3 and FT4 measurement at the first gestational check (18 weeks), THAb were presumably absent at that time of gestation (15). Likewise, in the present woman, in whom THAb were measured only 41 days postpartum, they were presumably absent at week 5 of gestation but present at week 20. Thus, considering these chronological data, we could infer that THAb appear only after the first trimester of gestation. Notably, the case reported here is the only one describing the transplacental passage of THAb (IgG-T3 and IgG-T4) causing the same inconsistent TFT pattern in the newborn.

Our work shows two main limitations. First, it was not until day 41 after delivery that we became aware of the history, so the crucial serum assay to demonstrate our hypothetical explanation about the TFT incongruence could not be performed in the previous blood samplings. Secondly, the newborn was not further monitored from the laboratory point of view in the following months as clinical signs suggestive of thyroid dysfunction never occurred. To partially overcome this latter limitation, the mother was monitored at 7 and 18 months after delivery, eventually demonstrating the return to a normal TFT pattern, suggesting the clearance of THAb. Regarding this last aspect, there are no detailed studies on the half-life of THAb. In a somewhat pertinent paper, published in 1997 on JCEM (13), the results reported a short half-life of THAb-IgM (from 15 to 30 days) and a longer one of THAb-IgG (of a few months), in line with the immunological theory according to which IgM class and Ig class have a half-life of a few days and of about 30 days, respectively.

In conclusion, this case represents clinical mismanagement of a pregnant woman with an abnormal TFT pattern, transmitted even to the newborn via the placenta. The increase of FT4 and FT3, discordant with the serum TSH in a clinically euthyroid pregnant woman, should have prompted physicians to suspect an analytical interference and, therefore, to interact with the laboratory in order to measure FT4 and FT3 with different analytical platforms and possibly TT4 and TT3 to better characterize the TFT pattern. In addition, since palpitations, dyspnea and tachycardia can be observed in healthy euthyroid pregnant women, the use of methimazole in these situations represents a potentially harmful overtreatment.

## Declaration of interest

The authors declare that there is no conflict of interest that could be perceived as prejudicing the impartiality of the research reported.

## Funding

This work did not receive any specific grant from any funding agency in the public, commercial, or not-for-profit sector.

## Statement of ethics

The study was conducted in accordance with the Helsinki Declaration. Written informed consent was obtained from the adult patient for both herself and her minor daughter. Information revealing subjects’ identity has been avoided. Both patients have be identified by aliases and not by their real names.

## Data availability statement

All data generated or analyzed during this study are included in this study. Further enquiries can be directed to the corresponding author.

## Author contribution statement

F D designed the idea, performed analyses, interpreted the results and drafted the manuscript; A B collected clinical data and drafted the manuscript; C C collected clinical data, discussed the results and approved the final manuscript; F G collected clinical data, discussed the results and approved the final manuscript; J Z performed albumin sequence analysis, discussed the results and approved the final manuscript; S M performed albumin sequence analysis, discussed the results and approved the final manuscript; F C supervised the analyses, discussed the results and approved the final manuscript; S B conceived the idea, performed analyses, discussed the results, revised critically the work and approved the final manuscript.
